# Gliosarcoma With Glioneuronal and Rhabdomyosarcoma Components

**DOI:** 10.7759/cureus.26695

**Published:** 2022-07-09

**Authors:** Murad Alturkustani

**Affiliations:** 1 Pathology, King Abdulaziz University, Jeddah, SAU

**Keywords:** rhabdomyosarcoma, tumor, ganglioglioma, glioneuronal, gliosarcoma

## Abstract

Gliosarcoma is a rare subtype of glioblastoma, isocitrate dehydrogenase (IDH) wildtype. This biphasic tumor has two components. The first one is glial and usually represented by glioblastoma. The second is a sarcomatous component usually represented by nonspecific spindle cell sarcoma. Rarely, different glial tumors could represent the non-sarcomatous component, including oligodendroglioma and ependymoma. There were only two reported cases in the literature with glioneuronal components (both were anaplastic ganglioglioma) as the non-sarcomatous component. This work reports a gliosarcoma in the right frontal lobe of a 13-year-old female with a glioneuronal tumor representing the non-sarcomatous component and a rhabdomyosarcoma representing the sarcomatous component. The child lived for only six months after the resection of the tumor. The short survival attests to the dismal prognosis of gliosarcoma regardless of the nature of the non-sarcomatous component.

## Introduction

Gliosarcoma is a rare subtype of glioblastoma, isocitrate dehydrogenase (IDH) wildtype, and it is considered a central nervous system (CNS) World Health Organization (WHO) grade 4 tumor [1]. It is common in patients aged 40-60 years and rare in children [2]. The biphasic neoplasm contains both glial and sarcomatous components. The most common glial component is glioblastoma, but IDH-mutant astrocytoma, oligodendroglioma (oligosarcoma) [3], and ependymoma (ependymosarcoma) could represent this component [4]. The possibility of ganglioglioma as the non-sarcomatous component was reported only in two cases [5,6]. The most common sarcomatous component is undifferentiated spindle cell sarcoma [1], while rhabomyoblastic differentiation is rare [7]. The commonly accepted theory for the origin of the sarcomatous component is a metaplastic differentiation from the gliomatous cells [1].

## Case presentation

A 13-year-old female patient was diagnosed with T-cell acute lymphoblastic leukemia with CNS involvement at the age of six years. She was treated with combined chemotherapy and radiotherapy. She was presented to the emergency department with a progressive left-sided weakness for three weeks and headache and vomiting for one week. The past medical history was significant for primary hypothyroidism, growth hormone deficiency, and primary ovarian failure.

**Figure 1:**
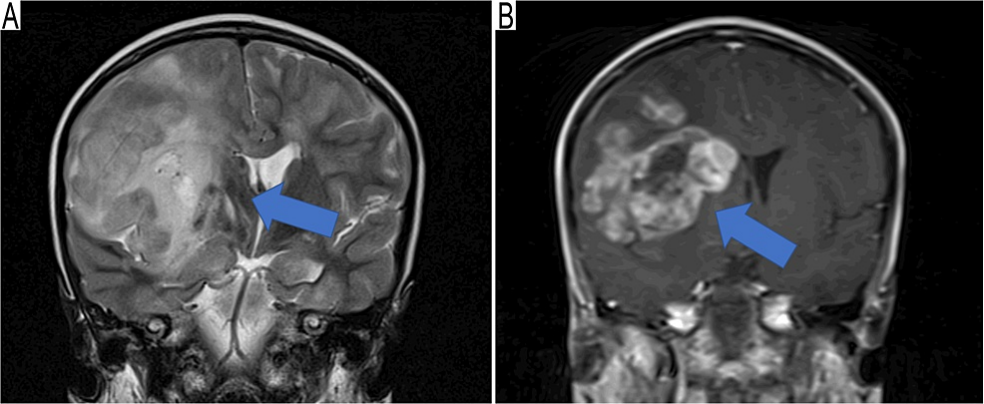
Imaging studies A predominantly right frontal mass. (A) T2-weighted image shows a heterogeneous high signal intensity (arrow) with minor surrounding edema. (B) T1-weighted image with contrast shows a heterogeneous pattern of enhancement (arrow).

Magnetic resonance imaging (MRI) examination of the brain showed a large (4.4 cm x 3.19 cm x 3.4 cm) right frontal mass with heterogeneous enhancement (Figure 1, Panels A and B). The differential diagnosis included infections and atypical leukemic infiltration.

**Figure 2:**
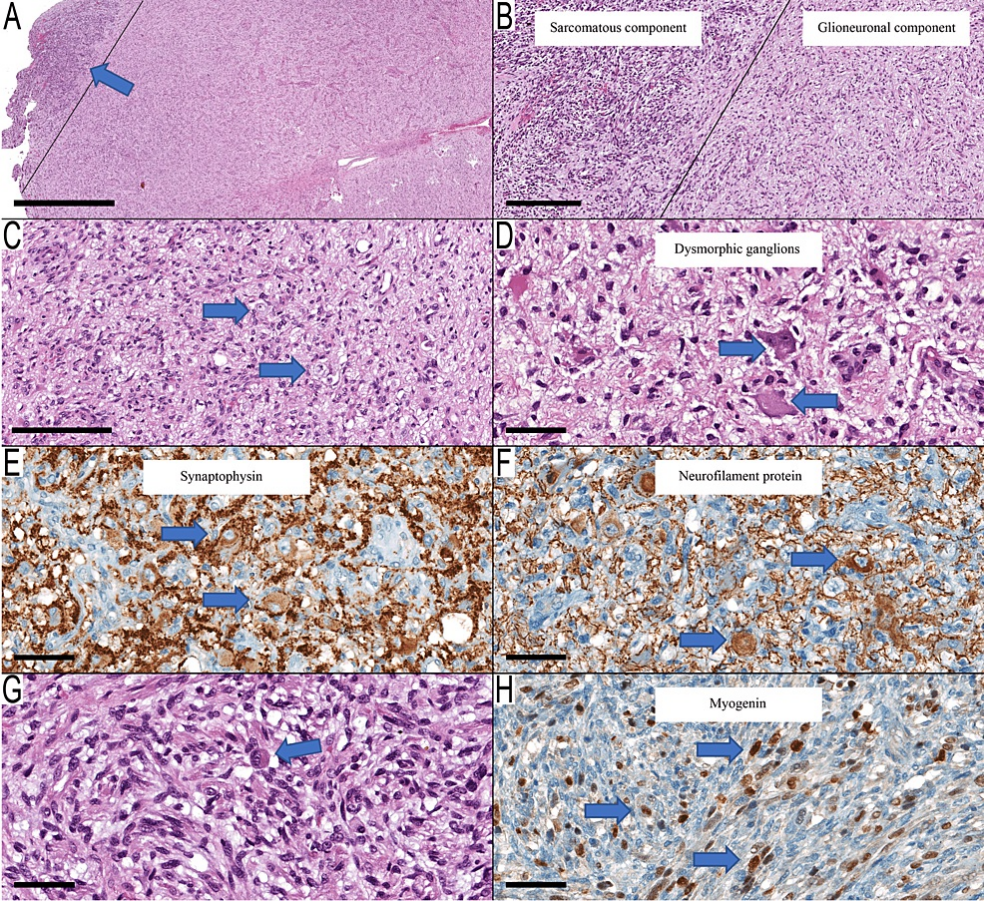
Pathological features of the tumor (A, B) A biphasic neoplasm is formed by a glioneuronal component (right side of the oblique line and arrow) and a smaller sarcomatous component (left side of the oblique line). (C, D) The glioneuronal component is formed by infiltrative elongated astrocytic cells with many dysmorphic ganglions (arrows). (E, F) Synaptophysin (E) and neurofilament protein (F) highlight the dysmorphic ganglions (arrows). (G) The sarcomatous component is formed by spindle cells with occasional eosinophilic cytoplasm (arrow). (H) Myogenin immunostain shows nuclear staining in the neoplastic cells (arrows) confirming their rhabdomyoblastic differentiation. Scale bar: 2 mm (A), 300 µm (B), 200 µm (C), 60 µm (D-H).

The mass was incompletely resected. Microscopic examination of the resected mass showed an infiltrative biphasic neoplasm (Figure 2, Panels A and B). The predominant component was formed by moderately cellular neoplastic astrocytes, few mitoses, and microvascular proliferation. Many dysmorphic ganglions were arranged haphazardly with scattered binucleation forms (Figure 2, Panels C and D). There was perivascular lymphocytic infiltrate but no eosinophilic granular bodies. The neoplastic glial cells were immunopositive for glial fibrillary acidic protein (GFAP). The dysmorphic ganglions were immunopositive for neurofilament protein (NFP) and synaptophysin (Figure 2, Panel E and F). IDH1 was immunonegative, and p53 showed scattered immunopositive nuclei. The second component, which represents the sarcomatous component, was formed by densely packed malignant spindle cells exhibiting nuclear pleomorphism and frequent mitoses. There were many rhabdomyoblastic cells with eosinophilic cytoplasm and occasional striations (Figure 2, Panel G). Myogenin immunostain showed nuclear staining in the rhabdomyoblastic neoplastic cells and many spindle cells in the sarcomatous component (Figure 2, Panel H). The proliferative index by Ki67 immunostain was 20% in the sarcomatous component, while it was 4%-5% in the glioneuronal component.

An accredited outside laboratory performed pan-cancer genomic profiling for the tumor using TruSight Oncology 500 (Illumina, San Diego, California). The result was reported as no significant alteration was detected. The integrated diagnosis was gliosarcoma with glioneuronal component and rhabdomyosarcoma. The patient was on palliative treatment and passed away six months after resection.

## Discussion

This case is the third reported gliosarcoma, up to my knowledge, with a glioneuronal tumor as the non-sarcomatous component. The non-sarcomatous component is difficult to classify in this case. The presence of infiltrative neoplastic astrocytes and dysmorphic ganglions indicated a glioneuronal tumor. Among glioneuronal tumors, anaplastic ganglioglioma would be the most likely possibility. The molecular test did not show evidence of mitogen-activated protein kinase (MAPK) pathway alteration. This alteration is an essential diagnostic criterion for an unresolved ganglioglioma [8]. However, not all possible MAPK pathway alterations can be detected by TruSight Oncology 500 testing, and the possibility of this pathway activation cannot be excluded. The differential diagnosis of this component is an infiltrative glioma with reactive ganglions. The presence of dysmorphic ganglions, especially with the binucleated form, favors the diagnosis of a glioneuronal tumor rather than infiltrative glioma. The sarcomatous component was sufficient for the gliosarcoma diagnosis regardless of the nature of the other component. The prognosis of gliosarcoma is similar to glioblastoma and, in some cases, even worse [9].

There are only two previously reported cases in which the non-sarcomatous component represents a glioneuronal tumor. The glioneuronal component in these two cases was considered an anaplastic ganglioglioma. Suzuki et al. reported a case of anaplastic ganglioglioma with an undifferentiated sarcoma component in a 59-year-old male. The ganglioglioma contained a classic low-grade area with a transition to another area with anaplastic features. The spindle cells in the sarcomatous component were immunopositive for smooth muscle actin (SMA) but immunonegative for desmin and cluster of differentiation (CD) 34. The sarcomatous component was considered fibrosarcoma or malignant fibrous histiocytoma. The clinical follow-up of the patient was not documented [5].

Zhi et al. reported another gliosarcoma case with anaplastic ganglioglioma in a 46-year-old male. The tumor was circumscribed solid and cystic in the left temporal lobe that extended to the optic chiasm and brainstem. The ganglioglioma component comprised dysmorphic ganglions and glioma with high-grade features (microvascular proliferation, significant mitotic activity, and necrosis). The sarcomatous component was formed by undifferentiated pleomorphic and spindle neoplastic cells and areas of pseudopalisading necroses. These cells were immunonegative for SMA, desmin, CD34, CD68, and epithelium membrane antigen (EMA) immunostains. Unfortunately, the follow-up was only three months after the surgery [6].

## Conclusions

This report is the third gliosarcoma case, to my knowledge, with a glioneuronal component representing the non-sarcomatous component and rhabdomyosarcoma representing the sarcomatous component. The most common glial component in gliosarcoma is glioblastoma (WHO grade 4), which could explain the poor prognosis in these tumors. However, in some cases, the prognosis is even worse than glioblastoma. The spectrum of the non-sarcomatous component is expanding to include tumors with lower WHO grades such as oligodendroglioma and ependymoma. This report expands the spectrum of these cases by confirming the possibility of a glioneuronal component (WHO grade 3) as the non-sarcomatous component. The bad prognosis, in this case, is similar to the prognosis of gliosarcoma with glioblastoma as the glial component. Future studies with more similar cases are required to confirm this bad prognosis.

## References

[1] Louis DN, Aldape KD, Capper D (2021). Glioblastoma, IDH-wildtype. Central Nervous System Tumours: WHO Classification of Tumours, 5th Edition.

[2] Karremann M, Rausche U, Fleischhack G, Nathrath M, Pietsch T, Kramm CM, Wolff JE (2010). Clinical and epidemiological characteristics of pediatric gliosarcomas. J Neurooncol.

[3] Rodriguez FJ, Scheithauer BW, Jenkins R (2007). Gliosarcoma arising in oligodendroglial tumors ("oligosarcoma"): a clinicopathologic study. Am J Surg Pathol.

[4] Rodriguez FJ, Scheithauer BW, Perry A (2008). Ependymal tumors with sarcomatous change ("ependymosarcoma"): a clinicopathologic and molecular cytogenetic study. Am J Surg Pathol.

[5] Suzuki H, Otsuki T, Iwasaki Y (2002). Anaplastic ganglioglioma with sarcomatous component: an immunohistochemical study and molecular analysis of p53 tumor suppressor gene. Neuropathology.

[6] Zhi L, Yang L, Quan H, Bo-Ning L, Ying-Jie L (2009). Anaplastic ganglioglioma with gliosarcoma component. Pathology.

[7] Khanna M, Siraj F, Chopra P, Bhalla S, Roy S (2011). Gliosarcoma with prominent smooth muscle component (gliomyosarcoma): a report of 10 cases. Indian J Pathol Microbiol.

[8] Solomon DA, Blumcke I, Capper D, Gupta K, Varlet P (2021). Ganglioglioma. Central Nervous System Tumours: WHO Classification of Tumours, 5th Edition.

[9] Zhang G, Huang S, Zhang J, Wu Z, Lin S, Wang Y (2016). Clinical outcome of gliosarcoma compared with glioblastoma multiforme: a clinical study in Chinese patients. J Neurooncol.

